# Impact of amputation level and vaulting on loading parameters during level ground walking

**DOI:** 10.33137/cpoj.v8i1.44416

**Published:** 2025-03-07

**Authors:** E Pröbsting, T Schmalz, M Bellmann

**Affiliations:** 1 Clinical Research and Services, Research Biomechanics, Ottobock SE & Co. KGaA, Göttingen, Germany.; 2 HAWK University of Applied Sciences and Arts Göttingen, Germany.

**Keywords:** Amputation, Lower Limb Amputation, Level Walking, Vaulting, Biomechanics, Gait, Transtibial, Transfemoral, Hip Disarticulation, Knee, Ground Reaction Force, Gait Analysis

## Abstract

**BACKGROUND:**

Previous studies show that during level walking, the load on the contralateral side increases with more proximal amputation levels. Furthermore, a typical compensation mechanism, vaulting on the contralateral side, may also influence the load. However, no study has compared the load applied to the contralateral side across more than two different amputation levels.

**OBJECTIVE:**

The objectives of this study were to analyze the biomechanical impact of different lower limb amputation levels and vaulting on the load applied to the locomotor system.

**METHODOLOGY:**

Gait data from 82 individuals with different amputation levels (44 transtibial (TT), 30 transfemoral (TF), and 8 hip disarticulation (HD)) were retrospectively analyzed in this study. Peak knee adduction, flexion and extension moments, vertical ground reaction force peaks, and force rates were statistically analyzed between different amputation levels and between two groups “TF with vaulting” and “TF without vaulting”.

**FINDINGS:**

As the level of amputation increases, walking speed decreases and asymmetry of stance duration increases. TF individuals with vaulting tend to walk faster than those without vaulting. The first peak of vertical ground reaction forces, the peak knee adduction and extension moments increase, and the peak knee flexion moments decrease with higher amputation level. The higher the amputation level, the curve of the vertical ground reaction force becomes significantly steeper during the first 5% of the gait cycle (GC). The first peak of ground reaction forces, the knee flexion, extension and adduction moments tend to be higher in TF individuals with vaulting.

**CONCLUSION:**

In summary, a higher lower limb amputation level can increase loading on the contralateral limb and contribute to a higher incidence of vaulting during gait. The effect of vaulting as a compensation pattern leads to an additional increase in contralateral limb loading.

## INTRODUCTION

During the rehabilitation process after a lower limb amputation, one of the most important goals is the restoration of standing and level ground walking. Several studies have investigated level walking in individuals with lower limb amputation^1–8^ and compared their gait to that of able-bodied individuals.^1,2,7,8^ Most of these studies have analyzed effects of different prosthetic components, mainly different prosthetic feet^3^ and prosthetic knee joints,^4,5^ on improving safety and mobility. A small number of studies also analyzed possibilities and limitations of different prosthetic hip joints.^6,9^

Studies have shown that gait asymmetry is common in people with lower limb amputation.^1,2^ Compared to able-bodied individuals, people with lower limb amputation generally walk slower, including a prolonged stance phase duration on their contralateral limb compared to the residual one and to able-bodied individuals.^1,8^ Nolan and Lees^7^ have shown that people with amputation compensate the loss of one or more joints by increased net joint moments and power output at their contralateral ankle, knee and hip joint compared to able-bodied individuals.^1,10^ Pröbsting et al., found no increased joint moments on the contralateral limb in people with TT amputation.^8^

The asymmetry in step length and stance phase duration increases with higher amputation level.^2^ However, the literature shows that individuals with TT amputation can still achieve a gait pattern similar to that of able-bodied individuals^1,2,7,8^ as they can actively control their knee joint,^8^ compared to those with TF and HD amputation.^5,6^ Studies state a more asymmetric gait, with prolonged stance phase duration and increased load on the contralateral side, in individuals with TF amputation compared to those with TT amputation.^7,11,12^ The latter explains the increased risk of contralateral knee joint osteoarthritis in individuals with TF amputation,^13^ as the increase in frontal and sagittal knee moments, and ground reaction forces, could contribute to development of knee joint degeneration in able-bodied individuals.^14–16^

Able-bodied individuals are able to control the distance between the foot and the ground (foot clearance) through the coordination of ankle dorsiflexion, knee joint flexion and hip joint flexion.^17,18^ Missing active dorsiflexion (in TTs, TFs, and HDs), active knee joint flexion (in TFs and HDs) and/or active hip joint flexion (HDs) affect foot clearance. Catching the ground with the prosthetic foot can result in a fall. In order to reduce the risk of falling, individuals with lower limb amputation develop compensatory strategies, such as vaulting, hip hiking and circumduction,^19^ which could lead to gait asymmetry.

Vaulting is the most prevalent method described by people with lower limb amputation and clinicians, yet it is not often discussed in the literature. Smith et al. described vaulting as “*a premature midstance plantar flexion by the contralateral limb which assists toe clearance of the prosthetic limb by lifting the body*”.^20^ In a big cohort of individuals with different lower limb amputation levels, vaulting occurred more frequently with higher amputation level,^2^ but no further analysis was conducted. Drevelle et al^21^ used quantitative gait analysis to evaluate vaulting motion pattern in individuals with TF amputation. Those who use vaulting as a compensatory movement exhibited a higher peak in generated power at the contralateral ankle during the contralateral single stance phase.^21^ Subsequently, vaulting and the level of amputation seem to influence the load on the contralateral side. To the knowledge of the authors, no study has yet analyzed the contralateral load for more than two different amputation levels and only one study has analyzed the influence of vaulting on the contralateral single stance phase.^21^ Therefore, the purpose of the present study was to describe the biomechanical effects of different amputation levels and vaulting on the loading parameters of the contralateral side, specially knee joint moments and vertical ground reaction force. The primary hypothesis was that as the level of amputation increases, the load on the contralateral side also increases during stance. The secondary hypothesis was that vaulting increases load on the contralateral side compared to non-vaulters between mid-stance to pre-swing.

## METHODOLOGY

### Data collection

Gait data from 82 individuals with different lower limb amputation levels were retrospectively analyzed in this study. Gait analyses have been conducted at Ottobock's gait lab in Göttingen since 2002. Gait data were captured using a VICON system (8 M-cams with measurement frequency 100Hz till 2013 and subsequent 12 Bonita cams (200Hz), VICON PEAK, Oxford, GB) coupled with two force plates (measurement frequency 1000Hz; Kistler 9287A, Winterthur, CH).

The study was conducted according to the declaration of Helsinki regarding human medical experimentation and entirely complies with the requirements of the German medical device act as well as the data protection law. Subjects gave their full verbal consent being measured and that pseudonymized data can be used for retrospective analyses and publication.

The biomechanical data used for this retrospective analysis were recorded from patients fitted in an orthopedic workshop. Measurements were taken at the end of the fitting process for documentation and quality assurance of the regular everyday fitting.

The following inclusion criteria were used in this study:

- Individuals with unilateral amputation (TT, TF (no short stump ≤ 1/3 of contralateral femur length) or HD).
- Age > 18 years.
- No additional health impairment.
- Able to walk at a self-selected velocity on level ground.
- Use of a commercially available Energy Storing and Returning (ESR) foot.
- TF prosthesis with Genium knee joint.
- HD prosthesis with C-Leg knee joint and Helix3D hip joint.
- Prosthesis aligned according to the criteria defined by Blumentritt^22^ for TTs and Bellmann for TFs^23^ and HD.^24^

Furthermore, the group of TFs was divided into two groups: “TF with vaulting”, and “TF without vaulting”. To make this differentiation, we used the method described by Drevelle,^21^ assuming that people with ankle flexion power values higher than 0.15 W/kg during single stance support conduct vaulting.^21^

### Data analysis

Three-dimensional marker trajectories were tracked from 17 markers placed on anatomical landmarks (both sides: acromion, epicondylus lateralis humeri, processus styloideus ulnare, trochanter major, compromise knee centre of rotation according to Nietert,^25^ malleolus lateralis, caput os metatarsale IV; and three asymmetric markers: left tibia, right thigh and left shoulder blade). This marker set has been used since 1998 and was created to analyze essential gait parameters for people with amputation.^8^ External joint moments were calculated based on ground reaction forces and coordinates of the joint centers as described in a previous study.^8^

In order to quantify the load on the contralateral leg, to test both hypotheses, the vertical ground reaction force and the external sagittal and frontal moments acting on the contralateral knee joint were evaluated.^14–16^ The first and second peak of the vertical ground reaction forces, the peak flexion and extension knee moments and the first peak of the frontal knee moments were identified and statistically analyzed.

Furthermore, the assessment of the force increase characteristic is a frequently used parameter to evaluate the load on the contralateral side.^26–29^ It is known from studies of running that a steeper increase correlates with a higher risk of injury.^26,29^ There are various analysis options for this parameter.^26^ In the present study, to test the first hypothesis, the increase of the vertical ground reaction force in the first 5% gait cycle (GC) was determined by the difference between the first value and the value at 5% GC. Moreover, spatiotemporal gait parameters were reported as well. All kinetic data were normalized to the stance phase of the gait cycle.

### Statistical analysis

Mean values for all parameters were determined based on 8 to 12 single gait cycles for the contralateral limb. Group means were calculated based on the values of all TTs, TFs and HDs and also for the two groups “TF with vaulting” and “TF without vaulting”.

The Kolmogorov-Smirnov test was used to analyze the normal distribution of the data. Afterwards, the Bartlett's test was used to identify whether equal variances exist. Since these two requirements were met, the differences in the peak values of biomechanical parameters and spatiotemporal gait parameters between the amputee groups were tested with the one-way ANOVA for each evaluated parameter. Post-hoc analyses with Bonferroni's corrections were performed when ANOVA showed significant differences. The significance level was set at p < 0.05. All analyses were performed using the WinStat software (Version 2012.1.0.96).

## RESULTS

### Participants

Data from 44 TTs, 30 TFs and 8 HDs were used for the analysis. Detailed information about the participants is shown in **Table 1**.

**Table 1:** Participant anthropometric data.

**Amputation level**	**TT**	**TF**	**HD**
	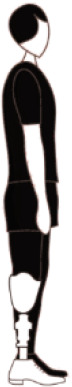 ESR	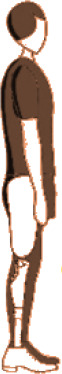 Genium ESR	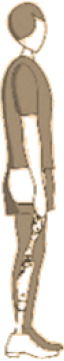 Helix C-Leg ESR
**Number of patients**	44	30	8
Age [Years]^*^	47 ± 15	45 ± 14	43 ± 12
Height [cm]^*^	176 ± 13	183 ± 5	175 ± 10
Weight [kg]^*^	88 ± 24	84 ± 9	76 ± 16
Vaulting rate^**^	12%	63%	100%

* Mean ± SD

** Determined based on the assumption that the ankle power on the sound side is > 0.15 W/kg.

The individuals with TT, TF, and HD amputation had an average age of 47 (SD=15), 45 (SD=14), and 43 (SD=12) years, respectively. Their average heights were 176 cm (SD=13), 183 cm (SD=5), and 175 cm (SD=10), while their respective weights, measured with the prosthesis, were 88 kg (SD=24), 84 kg (SD=9), and 76 kg (SD=16). Using the method of Drevelle,^21^ 12% of the TTs, 63% of the TFs and all HDs vaulted.

### Spatiotemporal gait parameters

As the level of amputation increases, walking speed decreases from 1.31 ± 0.17 m/s to 1.13 ± 0.16 m/s with no significant differences across all amputation levels. Vaulting TFs tend to walk faster than TFs without vaulting (1.28 ± 0.19 m/s vs. 1.22 ± 0.11 m/s). Likewise, the length of the stance phase decreases on the prosthetic side and increases on the contralateral side in correspondence with a more proximal amputation level. Only a few differences are statistically significant (**Table 2**). However, the asymmetry of the stance phase duration differed significantly between all amputation levels. The TTs show the lowest asymmetry with 2.7 ± 2.0% GC, while the HDs show the greatest with 9.3 ± 4.5% GC.

**Table 2:** Spatiotemporal gait parameters and statistical data for all three amputation levels and the two subgroups, “TF with vaulting” and “TF without vaulting”.

	TT	TF	HD	TF without vaulting	TF with vaulting	significant differences p<0.05
**Velocity [m/s]**	1.31 ± 0.17	1.26 ± 0.16	1.13 ± 0.16	1.22 ± 0.11	1.28 ± 0.19	Not significant
**Stance duration sound side [%gait cycle]**	64.3 ± 2.1	65.1 ± 2.2	67.6 ± 3.3	65.6 ± 1.8	64.9 ± 2.3	HD vs. TT HD vs. TF with vaulting
**Stance duration prosthetic side [%gait cycle]**	61.7 ± 1.4	59.3 ± 1.8	58.5 ± 2.2	59.9 ± 1.5	58.9 ± 1.8	HD vs. TT HD vs. TF HD vs. TF without vaulting HD vs. TF with vaulting
**Asymmetry stance duration sound side - Prosthetic side [%gait cycle]**	2.7 ± 2.0	5.9 ± 2.2	9.3 ± 4.5	5.6 ± 2.8	6.0 ± 1.9	TT vs. TF TT vs. TF without vaulting TT vs. TF with vaulting TT vs. HD HD vs. TF HD vs. TF without vaulting HD vs. TF with vaulting
**Step length sound side [m]**	0.69 ± 0.07	0.69 ± 0.06	0.71 ± 0.06	0.67 ± 0.05	0.70 ± 0.07	Not significant
**Step length prosthetic side [m]**	0.74 ± 0.08	0.74 ± 0.08	0.66 ± 0.08	0.73 ± 0.05	0.74 ± 0.09	Not significant
**Asymmetry step length sound side - Prosthetic side [m]**	−0.05 ± 0.05	−0.04 ± 0.06	+0.05 ± 0.07	−0.06 ± 0.05	−0.03 ± 0.07	HD vs. TT HD vs. TF HD vs. TF without vaulting HD vs. TF with vaulting

In terms of step length asymmetry, the HD group differs significantly from all amputation levels as well as from the TFs with and without vaulting. This group is the only one showing longer step lengths on the contralateral side than on the prosthetic side (**Table 2**).

### Vertical ground reaction forces

**Figure 1:**
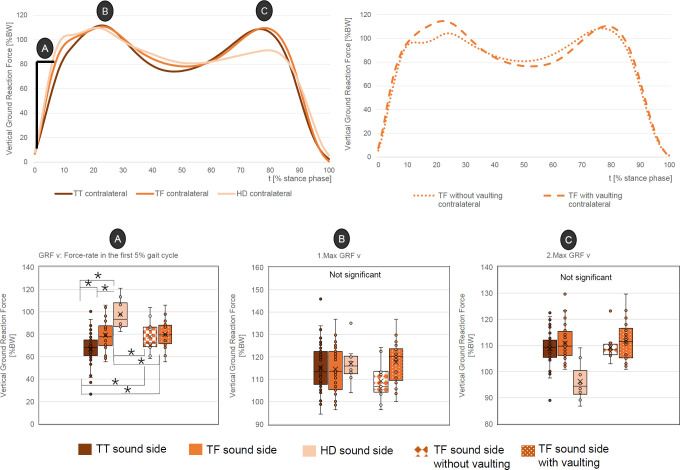
Top: Mean course of contralateral vertical ground reaction force (time normalized) for all three amputation levels (upper left) and the two subgroups, “TF with vaulting” and “TF without vaulting” (upper right). Bottom: Relevant peak values with statistical data.

The values of the first and second peak of the contralateral vertical ground reaction forces did not show any significant differences between the amputation levels nor between the two TF groups (**Figure 1**). However, there is a tendency that the first peak increases with a higher amputation level (TTs: 115 ± 11% BW, TFs: 115 ± 10% BW, HDs: 117 ± 9% BW) and also in the TFs with vaulting (118 ± 10% BW vs. 109 ± 9% BW).

In contrast, the force rate during the first 5% gait cycle differed significantly between all amputation levels. The higher the amputation level, the steeper the force rate (HDs: 98 ± 14% BW, TFs: 79 ± 14% BW and TTs: 67 ± 15% BW). In conclusion, the first peak was reached earlier with higher amputation levels.

### Contralateral sagittal knee moments

**Figure 2:**
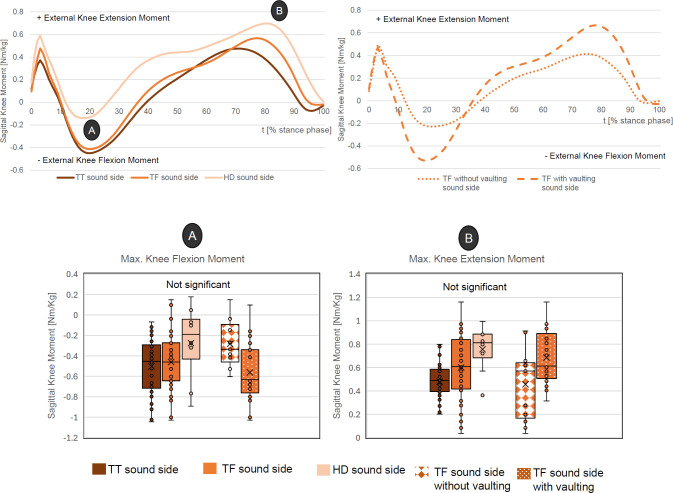
Top: Mean course of external contralateral sagittal knee moments (time normalized) for all three amputation levels (upper left) and the two subgroups, “TF with vaulting” and “TF without vaulting” (upper right); Bottom: Selected peak values with statistical data.

In the HDs group, there was a general trend towards increased extension moments on the contralateral knee compared to TTs and TFs. As the amputation level increased, the peak flexion moments tend to decrease, (TTs: -0.50 ± 0.28 Nm/kg, TFs: -0.46 ± 0.30 Nm/kg, HDs: 0.27 ± 0.38 Nm/kg), whereas the peak extension moment increased (TTs: 0.51 ± 0.19 Nm/kg, TFs: 0.60 ± 0.29 Nm/kg, HDs: 0.76 ± 0.20 Nm/kg). The TFs without vaulting showed a reduced peak flexion moment (-0.28 ± 0.24 Nm/kg vs. -0.56 ± 0.29 Nm/kg) and a reduced peak extension moment (0.46 ± 0.32 Nm/kg vs. 0.68 ± 0.24 Nm/kg). All differences are statistically not significant (**Figure 2**).

### Contralateral frontal knee moments

**Figure 3:**
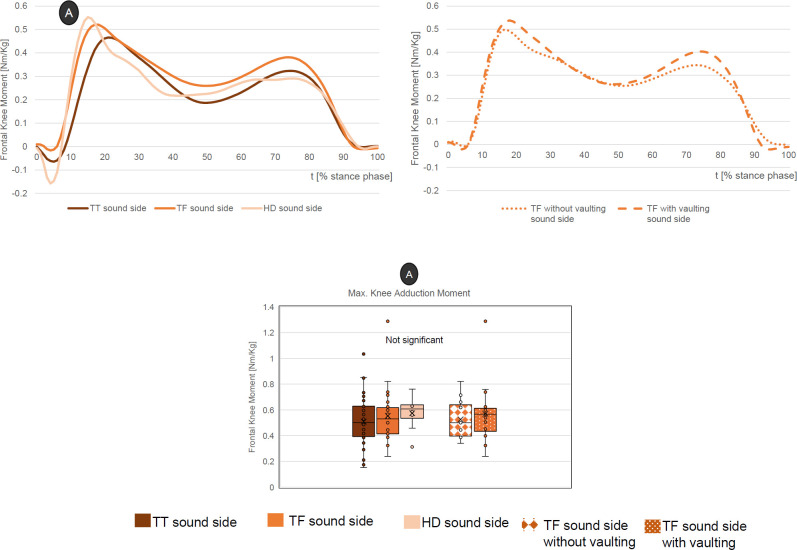
Top: Mean course of external contralateral frontal knee moments (time normalised) for all three amputation levels (upper left) and the two subgroups, TF with vaulting” and “TF without vaulting” (upper right). Bottom: Selected peak values with statistical data.

Generally, the peak adduction moment (first peak for all subjects) increases with a more proximal amputation level (TTs: 0.51 ± 0.19 Nm/kg, TFs: 0.55 ± 0.20 Nm/kg, HDs: 0.57 ± 0.14 Nm/kg). The TFs with vaulting also show an increased peak compared to those without vaulting (0.57 ± 0.22 Nm/kg vs. 0.53 ± 0.16 Nm/kg). Nevertheless, none of these differences show statistical significance (**Figure 3**).

## DISCUSSION

The objectives of this study were to analyze the biomechanical impact of different lower limb amputation levels and vaulting on the load applied to the locomotor system. The higher the amputation level, the curve of the vertical ground reaction force becomes significantly steeper during the first 5% GC. The first peak of vertical ground reaction forces, the peak knee adduction and extension moments tend to be higher both in individuals with higher amputation level and in TF individuals with vaulting.

Peak knee flexion moments decreased with higher amputation levels and tended to be higher in TF individuals with vaulting. Individuals with lower limb amputation have an increased risk of developing knee joint degeneration.^13^ Therefore, any increase in knee joint loading forces and moments is clinically relevant. For this reason, the nonsignificant increases in forces and moments observed in this study, are still clinically important.

***Primary hypothesis:*** Load on the contralateral side increases with higher amputation level.

During the first 5% gait cycle, the vertical ground reaction force increased significantly faster with a more proximal amputation level, although the walking speed decreased accordingly. This interrelation between force rate and walking speed is untypical. Typically, the increase in vertical ground reaction force becomes steeper as walking speed increases.^30^

The prosthetic limb exhibited a reduced stance and prolonged swing duration. As noted by Ding et al.,^31^ this may contribute to a more abrupt “landing” on the contralateral limb, evidenced by a significantly greater peak knee loading rate and a significant increased force rate observed in this study. Subsequently, the first peak of vertical ground reaction force was increased with higher amputation level. This, in turn, impacted the first peak knee adduction moment, which was also slightly increased with higher amputation level, but more pronouncedly. Based on these effects, it can be concluded that knee compression forces were also increased.^32^ Thus, the shortened prosthetic stance phase contributed to an increased load on the contralateral side. This should be avoided by an optimally aligned prosthesis, adequate prosthetic components and appropriate gait training. Increased knee extension, or rather less knee flexion moments, were identified for higher amputation levels, both at the beginning and at the end of stance. Although these peaks are not significantly different, a clear and systematical tendency can be observed with a higher amputation level. The missing significance might be in relation to the speed differences within each group.

All individuals in this study, independent of their level of amputation, showed a prolonged stance phase duration on the contralateral side compared to the prosthetic side. This leaded to an asymmetric stance phase duration, as confirmed by other studies comparing TTs and TFs with able-bodied individuals^1^ and with each other.^7^

The results of the present study confirmed the results of Heitzmann et al.^2^ and showed that asymmetric stance phase duration significantly differed across all amputation level, with TTs showing the smallest and HDs showing the greatest asymmetry. Therefore, the contralateral limb experienced comparatively higher loading over a significantly longer time with a higher amputation level. Thus, it can be assumed that the significantly longer loading duration, combined with the significant faster load transmission and higher ground reaction forces, as well as sagittal and frontal moments, explain the higher prevalence of knee osteoarthritis^13^ with higher amputation level.

Consequently, the primary hypothesis of the present study stating that a more proximal amputation level increases the load on the contralateral side is confirmed by the results.

***Secondary Hypothesis:*** Vaulting increased the load on the contralateral side.

In comparison with able-bodied individuals, prosthesis users experience a reduced ability to actively control the prosthesis with the residual limb as the level of amputation increases (from TT to HD), and their strategies for achieving ground clearance become more limited. As a result, the contralateral side is used more intensively for compensation with vaulting being the most obvious and specific strategy. This strategy was observed in all HDs, 63% of the TFs and only 12% of the TTs in the present study. Increased vaulting with higher amputation level is a logical and sometimes necessary consequence and was already observed by Heitzmann et al.^2^

Some highly functional and safe prosthetic components, e.g. more functional hip joints,^6^ microprocessor knee joints^33^ and hydraulic ankle joints,^3^ support the generation of more ground clearance. But one of the main influencing factors is prosthetic alignment, specifically the anterior-posterior position of the knee joint axis of rotation and the foot.^33^ Furthermore, people with lower limb amputation rely on the ground clearance provided by the prosthesis without any sensory feedback. The prosthesis may not perform optimally especially in unpredictable situations and therefore patient-initiated vaulting is understandable. Besides contralateral forefoot pain, this compensation strategy could lead to biomechanical consequences, such as higher energy consumption than able-bodied individuals and an asymmetric loading distribution, with increased ground reaction forces on the contralateral limb.^21^ Furthermore, vaulting can also lead to increased ankle, knee and hip moments on the contralateral limb at the end of stance phase.^7^

For a more detailed analysis of the impact of vaulting on the loading of the locomotor system, the TF patient group was divided into two groups with and without vaulting. A similar separation was not useful for the other amputation levels. In the HD group, all individuals performed vaulting. In the TTs, the group with vaulting was too small compared to the “non-vaulting” group.

The following analyzed peak values were increased for the TFs with vaulting: first and second peak ground reaction forces, first peak knee adduction moment, max. knee flexion and max. knee extension moment. The most obvious difference between the two groups was found in the sagittal plane. On the one hand, TFs with vaulting showed more stance flexion,^21^ which could explain the higher knee flexion moments in the first part of stance. On the other hand, the faster anterior movement of the Center of Pressure (COP)^21^ and the prominent extension of the knee joint at the end of stance^21^ could explain the increased extension moments.^7,21^

Generally, walking speed influences the magnitude of joint moment peaks,^34^ and the TF group with vaulting walked 0.06 m/s faster than non-vaulting group. According to Lelas et al.,^34^ this speed difference could explain an increase in the peak knee flexion moment by 0.02 Nm/kg and the peak knee extension moments by 0.003 Nm/kg. However, the difference in the peak knee flexion moment between the two groups of TFs was 0.20 Nm/kg and the difference in the peak knee extension moment was 0.22 Nm/kg, both of which were more pronounced. Therefore, the increase of knee joint moments for TFs with vaulting can be clearly attributed to this compensatory motion. Nevertheless, the secondary hypothesis that vaulting influences the load on the contralateral side can be confirmed to a certain extent as the results were not statistically significant, but were clinically relevant for patients showing unusually high loads.

### Limitation

The limitations of this study were, on the one hand, the different number of subjects with the respective amputation levels. The small number of people with HD amputation was a particular limitation in this study. On the other hand, determining the effect of vaulting was challenging when comparing two different cohorts of people with TF amputation, as individual knee moment heights were highly subject-specific. In order to reduce the latter limitation, an analysis of able-bodied individuals walking with and without vaulting should be conducted in future studies. This could provide a more detailed understanding of the specific effect of vaulting on knee joint moments.

## CONCLUSION

In summary, the loading of the contralateral limb increases with higher amputation level. The increase of knee loading is caused by the reduced stance duration of the amputated side with a fast force transmission onto the contralateral side in the first phase of stance. The effect of vaulting as a compensation pattern leads to an additional increase of contralateral limb loading. As the level of amputation increases, the ratio of vaulting in amputees increases, which means that the contralateral load also increases with the level of amputation. Therefore, the aim of gait training, prosthetic alignment and the selection of prosthetic components should support a more symmetric gait without or with only moderate compensatory vaulting patterns.
